# Technical Blossom in Medical Care: The Influence of Big Data Platform on Medical Innovation

**DOI:** 10.3390/ijerph17020516

**Published:** 2020-01-14

**Authors:** Bai Liu, Shuyan Guo, Bin Ding

**Affiliations:** 1 Business School, Jilin University, Changchun 130012, China; m18443152025@126.com; 2 International Business School Suzhou, Xi’an Jiaotong-Liverpool University, Suzhou 215123, China

**Keywords:** medical innovation, public health, big data, medical innovation investment, medical innovation patent

## Abstract

Medical innovation has consistently been an essential subject and a source of support for public health research. Furthermore, improving the level of medical research and development is of great concern in this field. This paper highlights the role of big data in public medical innovation. Based on a sample of China’s listed firms in the medical industry from 2013 to 2018, this paper explores the exogenous shock effect of China’s big data medical policy. Results show that the construction of the medical big data platform effectively promotes innovation investment and the innovation patent of medical firms. In addition, the heterogeneity of this promoting effect is reflected in firm size through the overcoming of different innovation bottlenecks. The research conclusions support the positive significance of the macro-led implementation of the medical big data platform, and suggest that the positive economic externalities generated by this policy are critical to public health.

## 1. Introduction

Public health innovation is a matter of concern to all humans. Compared with individual, customized treatments, the most critical significance of public health in medical research lies in the maximization of knowledge contribution and the extensive potential benefits [1]. Based on its widespread influence, medical innovation plays a key role in the development of public health, which has gotten extensive attention from academia for decades [2,3,4,5,6,7]. Nevertheless, when researchers further examined the research and development (R&D) situation of world health, they found that a lack of innovation for nontechnical reasons (resource preference, lack of funding, mismatch of costs and benefits) was very common, which profoundly affected the overall health level and sustainable development ability of local residents [8,9].

Many articles have pointed out related reasons for these circumstances. Although medical research itself can extend to a relatively mature and stable level, the for-profit nature and competition among medical enterprises have created a ceiling on R&D efficiency, proving that the introduction of medical technologies requires macro guidance [10,11]. Viergever suggests that the dependence of health R&D on market incentives has resulted in a global health R&D landscape, neglecting certain products and populations, and showing a distribution that is not “needs driven” [12]. With this imbalanced medical innovation, health is available in the majority of areas, but it has not been realized as “public” yet. In the current stage of scientific development, researchers still struggle to determine how to make public health more public. Many articles have suggested that the key to the solution lies in the financial assistance from philanthropists or governments, which can cover the developmental cost of such drugs or treatments in stages [13,14].

The reallocation of medical investment mainly focuses on financial providers. However, the positive externalities of technological change can bring underlying disruptive changes to public health, which is also worth exploring. Based on the impact of big data medical treatment in China, this paper proposes a new perspective for improving public medical innovation, which means that the cost reductions and R&D ascension that result from the data technological advances with the proper guidance of policies will support the broader medical research and development.

The mechanism to improve medical innovation is always a key issue in public health research. In recent years, with the influx of big data and artificial intelligence, more and more studies have been initiated on the impact of technological change, most of which are focused on the fields of basic medicine and public health [15,16,17]. The explosive growth of medical research as a result of the accumulation of knowledge and data has resonated not only with academics, but also with the funders of research and development [18]. Research which is related to life sciences shows that the integrity of data is of great significance to the judgment of material properties, as well as the development of materials, and big data improves the accuracy and reproducibility of research [19]. It also indicates that the cost reduction and efficiency improvement brought about by comprehensive data make it possible to conduct some experiments that were already expensive. As a result, some related databases are also gradually built and improved [20]. Moreover, other articles have predicted and evaluated the efficiency and output of big data in medical treatment, and asserted that the massive acquisition and extraction of information from both personal and public data could reduce the cost of research and development in the field of life sciences by increasing its output, speed and scalability, which will be of great significance in preclinical research and development [21].

Previous studies have specifically stated that big data is beneficial to the research and development of pharmaceutical companies [16,22,23]. Nevertheless, some articles raise questions about this subject. Verbrugghe and Colpaert [24] used a multi-center retrospective database to rethink the article of Cordier et al. [25]. They also proposed new insights into the established method of patient database management and the existing confusion regarding its practical application; that is, they explore whether the nonstructured, heterogeneous data contains insufficient information in the application of big data. Based upon the practical application, research has also revealed that, in the case of both big data healthcare and medical data, there are great differences in the standard assessment in the acquisition of primary medical data, which is inevitably one of the most critical and significant costs [26].

Due to the difficulty of obtaining experimental data in experimental sites, the existing literature has also cited actual clinical data for research, but it mostly focuses on how one can improve the process design, data privacy characteristics, data coding, etc., and does not yet verify the actual implementation effect of big data policy. For example, Lee et al. [27] mainly rely on the method of comparative analysis to investigate the policies and systems of different countries in protecting the privacy rights of big data medical treatment. Their work puts forth policy suggestions through the research. While there is also research focused on how data can be correlated to understand their own growth trends and tap into the potential for detail [28]. Some studies also concentrated on how to use program analysis to improve the effectiveness of big data in decision making and specifically covered big data in healthcare [29]. Detailed research, application and trend control are constantly discussed, and qualitative analysis of the impact of big data from a macro perspective is still scarce.

In summary, the conflict and mismatch between theoretical deduction and the application effect is a growing concern in the field of public health research [29,30,31]. Furthermore, the application of medical big data is generally scattered. Without government intervention, data resources cannot be developed substantially, so the advantages and disadvantages of any large-scale application of big data cannot be fully and scientifically tested. From the perspective of medical progress, does the application of big data in the medical field show valuable effects and returns? This article sheds light on the test of the actual effect of policies, and aims to fill the gap between theoretical deduction and practical application to some extent.

Based on the advantage of promoting the medical big data process by examining the context of provinces as units in China, special data applied in this paper can be obtained for exploration.

To our best knowledge, few actual studies have been conducted to verify whether this is the case, or whether this impression represents the “Productivity Paradox” of big data. However, such findings are crucial to the direction of the application of data technology. Its practical application value is undeniably crucial to the application of big data in the future medical field. The key to verifying the implementation effect of big data lies in the impact of exogenous events. Due to the data privacy and the lack of sharing caused by the competition of medical institutions, pharmaceutical companies must rely on policy guidance or macro arrangements to obtain big data information. Considering health care in the United States as an example, multiple stakeholders, including the pharmaceutical and medical product industries, suppliers, payers and patients, have different interest motivations, because their data pools are often disconnected due to competition. This leads to the uncombined use of clinical data, with which exogenous interventions are particularly important. Can exogenous intervention achieve the desired effect? This article answers this question based upon China’s medical big data policy. The design of this article provides data support to address this gap.

China is a unique country in the field of medical and health care. Many Chinese provinces offer experimental fields for the reform of the medical system. First, the institutional and cultural differences between Chinese provinces are smaller than in most other countries, meaning that the traits of the experimental and control groups are more similar, making China a good place to test the effects of pilot policies. Secondly, China is still a developing country, and it constantly tests feasible development methods. Due to this testing system, the implementation speed in China’s healthcare field is very fast, so the inspection caused by exogenous shocks is not easily disturbed by noise. Third, China has a large population and a high proportion of self-use in medical research, which can better reflect the positive externalities of the implementation of a research data medical policy on public health. Based on the above reasons, we chose China as the main research area, and further selected China’s big data medical pilot policy in eastern China in 2016 as the main case study.

On 21 October 2016, the National Health and Family Planning Commission of China selected two provinces (Jiangsu and Fujian) in eastern China as the development pilot areas for medical big data in which to establish an interconnected big data medical platform. This major step in the construction of China’s medical system provides a practical example for us to test the application effect of big data. Based on this exogenous impact and the available samples, this paper uses actual data to test the impact of the big data institutional arrangement on innovation in pharmaceutical enterprises and to determine whether the introduction of big data supports more positive assertions or whether it offers a new “Productivity Paradox”.

The results of this paper supplement the gap in the existing research and verify the application effect of medical big data from the perspective of society. The result shows that the big data medical policy has positive significance for promoting the innovation of medical enterprises, and this effect is heterogeneous for enterprises of different sizes. Among them, the positive effect of big data on small enterprises is mainly reflected in risk avoidance (due to comprehensive and accurate prediction), so as to increase the willingness of small enterprises to engage in research and development. For large enterprises, the positive role of big data mainly lies in making up for the lack of data resources, so it plays a greater role in R&D patent types. The results provide theoretical support for public health research and better reference for institution formulation.

In summary, this paper offers the following contributions. First, its framework contributes to the extant literature by introducing the actual effect of big data construction on medical enterprises, and enriches the research framework of medical big data. The research shows that the introduction of big data technology can reduce costs, improve overall efficiency, and address the current insufficient drug research and development in developing countries and regions. The second area in which this paper contributes is application. By exploring the implementation effect of pilot policies, this paper puts forward suggestions for the further promotion and application of big data medical treatment.

The authors use real public health data to verify the positive impact of big data medical system construction on medical research and development, and provides references for the implementation of relevant policies and governmental guidance. Furthermore, in view of the heterogeneity of enterprise size, the paper offers suggestions regarding different policy implementation and management innovation strategies.

The structure of this paper is as follows: The second section contains the research background and hypotheses of this paper; the third section introduces the methodology, sample and data, variables and research models; the fourth section introduces the empirical results, including descriptive statistics, parallel trend verification and regression results; and the fifth and sixth sections contain the research discussion and conclusions.

## 2. Background and Hypotheses

### 2.1. Big Data

With the development of information technology and the explosion of data, more and more studies are focusing on the application of big data in the medical and public health fields [15,17,19]. With the continuous penetration of the concept of big data, the academic circle has engaged in a profound discussion on its concept in recent years. Compared with data generated in traditional form, big data has three main characteristics: Volume, Variety and Velocity [32]. These “three Vs” represent high data volume, high data heterogeneity, and high data generation speed. The arrival of the era of big data has played an important role in industrial productivity and progress. A lot of productivity development and research is based on data, and today’s data structure has taken a qualitative leap from where it was a few years ago. This change in the nature of data sets may bring disruptive changes to the research of many disciplines [22].

Big data has attracted extensive attention in the medical industry, and this trend lies in the rapid digitalization of large numbers and the improvement of healthcare efficiency, including clinical decision support, disease surveillance and population health management, but there is still no accurate conclusion on its specific effects in the medical arena [33,34,35]. Big data medical operation ability in each level of granularity, privacy, security and quality assurance has seen development in the field regarding their difficulty, but inspection data in the medical field also brings challenges. Considering the comprehensive nature of public health care and popularity, one must consider several questions that arise [36,37]. Therefore, we suggest that the development of data technology and the guiding effect of macro policies will provide new solutions to this dilemma and realize more extensive public health advancement through reducing R&D costs and improving efficiency.

### 2.2. Big Data and Medical Innovation

In recent years, many studies have focused upon the impact of big data on medical and public health. According to the research by Dwivedi et al. [23], the increasing number of patients puts increasing pressure on the medical system, and makes it more and more difficult for patients to gain access to the primary medical staff. However, the Internet of Things (IoT), based on big data, can solve this problem well [23,38]. Hemingway et al. argue that, through big data technology, millions of health records, gene sequencing, imaging and other components of data, can build an expanded health digital trajectory to provide more convenient support for the actual clinical treatment [39]. The research by Sodhro et al. further shows that the combination of existing big data and computers to monitor data and provide medical services remotely has and will continue to have a positive and significant effect [40]. In addition, many other works of literature have proved the role of big data and algorithms in precision medicine [41,42].

To our best knowledge, the verification of the application results of big data in the existing papers is mostly focused on fine medical services, such as the effect of technology introduction on the average number of hospital visits and the efficiency of diagnosis and treatment, and the impact of detailed and continuous storage of patient files on the accuracy of diagnosis and treatment.

Although the improvement of precision medicine through big data has a profound impact on public health, this effect still misses the wider audience. For underdeveloped areas or low-income groups, medical services in hospitals face high barriers to entry, including consumption barriers and regional barriers. By contrast, the development and use of drugs is aimed at a wider audience, and is important for raising the threshold of public health. Literature suggests that the whole process of drug discovery and development includes target identification, hit discovery, hit-to-lead generation, lead optimization and pre-clinical drug candidate identification [16]. According to the existing literature, prescription drugs that take about 10–17 years and nearly 2.6 billion dollars to develop have less than a 10% approval success rate [43,44]. The high-risk, high-cost, long cycles and competitive nature of drug development inevitably lead companies to consider patients’ ability to lower the cost of research and development, resulting in the absence of much-needed drug development in some developing regions, of which the phenomenon has been realized through the advancement of computer-aided technology [45].

The basic theoretical literature and survey results also reveal that the arrival of big data has had a positive impact upon drug R&D. Cochrane and Galperin believe that data construction plays a fundamental role in supporting and promoting the basic research of life science [20]. Zhu makes suggestions from the perspective of technology [46], and asserts that massive data sets of drug candidates and the diverse clinical responses of patients indicate that modern drug research has entered the era of big data. Deep learning and big data modeling analysis will enable researchers to obtain new solutions for drug safety and efficacy evaluation. Moreover, the accumulation of a large amount of chemical and biological data gives the technology of computer-aided drug design an increasingly prominent role in the application, which is reflected in the advantage of rapid drug discovery and development; this can reduce costs and improve efficiency [16]. In summary, we believe that the big data medical platform will be conducive to R&D and the optimization of medical enterprises, which will have a significant impact on the drug discovery process, and ultimately affect public health.

Based on the above analysis, this paper puts forward the hypotheses:

**Hypothesis** **1.** The Big Data Medical System has a positive impact upon the innovation investment of medical companies.

**Hypothesis** **2.** The Big Data Medical System has a positive impact upon the innovation patents of medical companies.

## 3. Materials and Methods

### 3.1. Research Methodology

The methodology of this paper is quasi-natural experimentation, which is the core methodology in policy testing [47,48]. In contrast to the advance control of external conditions in medical and biological experiments, the experimental objects of quasi-natural experiments exist objectively in real life, and the tested policies actually occur in the public domain, so there is a higher level of validity when the policy effectiveness is tested and the scheme is proposed. In quasi-natural experiments, the Difference in Differences (DID) method is widely studied and has been proven in statistics; thus, this paper chooses the DID method as the specific empirical method [49,50].

The approach of DID applies to problems of multiple subgroups. Some are in the policy implementation group (the group subject to the intervention), while others are in the policy control group (the group not subject to treatment). Researchers examined the effects of policies qualitatively and quantitatively by comparing the results measured in each group before and after the policy intervention [51,52,53]. To account for time trends unrelated to the intervention, the change experienced by the treatment group is adjusted by the change experienced by the control group [54].

Due to the privacy and extensiveness of data, the establishment of a big data platform requires the formulation of macro policies, which formulation provides a suitable exogenous shock condition for testing the effect of big data. This paper carries out the research accordingly. The implementation of the medical big data pilot policy in China can be constructed as a quasi-natural experiment. On 21 October 2016, the National Health and Family Planning Commission officially issued a notice making Fujian and Jiangsu Provinces in eastern China the first batch of pilot provinces. It stated that China’s health and medical big data had officially entered the implementation stage. In order to verify the impact of big data on medical research and development through the research method of the quasi-natural experiment, we introduced the method of Difference in Differences. First, through the implementation of the policy, we divided the samples into the treatment group and the control group. If the province in which the firm is registered is a pilot province of big data medical treatment, then the sample of the firm is classified as the treatment group. Otherwise, it is classified as the control group. By comparing the innovation of the treatment group and the control group before and after the experiment, we verified the implementation effect of the policy.

As the “pioneers” of the national health and medical big data center, Fujian and Jiangsu Provinces base the collection on their own advantages and collect and integrate big data sources. The pilot provinces have gathered big data from municipal hospitals, county-level hospitals and other medical institutions, and gathered public health data, clinical data, genetic data, the Internet of Things (IoT) data, and nearly 10 billion pieces of data. On the premise of respecting and protecting privacy, the pilot areas will provide services in data, application, scientific research, ecology and security.

### 3.2. Sample and Data

This article mainly intends to study whether the medical big data policy promotes the innovation of medical enterprises. Considering three reasons, this paper choses the listed firm as the subject of the research sample. First, there is a certain scale barrier to the application of technology. Public companies tend to be more mature and are more likely to break through this barrier, which can better verify the implementation effect of this test. A-share listed companies work in line with this feature. Second, considering the availability of data, listed companies must regularly disclose data in accordance with the provisions of the China Securities Regulatory Commission, while non-listed companies do not have such a mandatory requirement, and voluntary disclosure may lead to the problem of sample selection bias. In summary, we chose China’s Shanghai and Shenzhen A-share listed companies as the subjects of the initial research samples. In the screening process, we only kept the data of medical companies from 2013 to 2018 (the classification methods are described in the next paragraph), and we excluded the operation abnormal data (return on total assets less than 0, asset-liability ratio more than 1 or less than 0, listed companies during that year). Finally, we considered that the medical big data pilot provinces are in eastern China. In order to ensure that the properties of the experimental group and the control group are more similar, we keep only the companies registered in east China.

In the field of medical enterprise, we refer to the Chinese standard classification industry in 2012, and through the manual screening, the trade name for medicine manufacturing, research and experimental development, and the health of the firm. Furthermore, the name of the firm and the main business scope includes medical, medicine, genes, cells, blood and diagnosis in the firm selected as a sample. Since the pilot policy of medical big data in 2016 was only implemented in Fujian and Jiangsu provinces in eastern China, in order to control the heterogeneity of environmental factors in quasi-natural experiments, the authors reserved listed companies in eastern China for research. It should also be noted here that since Shanghai is a municipality directly under the central government and Taiwan is an autonomous region, the basic situations in those two regions are different from those of other provinces, so this paper also excludes the firms of these two provinces. After the above sorting and screening, the final data of this paper includes 613 firm-year samples of Chinese A-share listed companies. These firms are all registered in eastern China (the provinces are: Jiangsu, Zhejiang, Anhui, Fujian, Shandong and Jiangxi). The raw data came from the CSMAR database in China, and the data sorting as well as empirical regression process used Excel and Stata 15.0 software.

### 3.3. Variable Definitions

#### 3.3.1. Dependent Variables

This paper aims to verify the impact of the application of medical big data on medical R&D. The existing articles present two main ways to measure innovation: investment and patent [55,56,57]. In this article, the construction of the big data platform has provided sufficient data support for pharmaceutical research and development, which has accelerated the process of market demand, targeting, application experiment, and feedback improvement in pharmaceutical innovation. This effect is reflected in both the firm’s innovation investment and its innovation patents. Innovation investment focuses on the willing perspective, and it is measured as the natural logarithm of R&D expenditures disclosed in the financial statements. Innovation patent focuses on the action perspective, which is measured by the number of patents granted during the fiscal year.

#### 3.3.2. Independent Variables

Through the implementation of the policy, we divided the samples into the experimental group and the control group. If the province where the firm is registered is the pilot province of big data medical treatment, then the sample of the firm is classified as the experimental group. Otherwise, it is classified as the control group. By comparing the R&D performance of the experimental group and the control group before and after the experiment, we verified the implementation effect of the policy. As the method adopted in this paper is the Difference in Differences (DID), the intra-group difference in time and the inter-group difference under impact both need to be compared simultaneously. Therefore, the explanatory variable DID in this article is the product value of the two variables Treat and After. First, define the variable “Treat”. If the province where the sample firm is located is the pilot area of medical big data, Treat = 1; otherwise, Treat = 0. Second, define the variable “After”. When the sample data is after the policy implementation, After = 1; otherwise, After = 0. Third, define the variable “DID”. When Treat = 1 and After = 1, DID = 1; otherwise, DID = 0. The definition of the explanatory variables in this paper as above.

#### 3.3.3. Control Variables

In the research, we control for the variables other than any independent variables that can cause changes in firm innovation, so as to make the results of quasi-natural experiments more accurate. Referring to the related literature of previous researches on firm innovation, this paper mainly selects the variables of basic firm characteristics, operating conditions and firm governance as the control variables.

Basic characteristics of the medical company have an impact on the innovation situation. In light of this, we selected Size, Lev and Age as our control variables. Size refers to the size of a company, measured by the natural logarithm of its total assets, plus one. Lev (Leverage) represents the company’s debt level, as measured by dividing the company’s total liabilities by its total assets. Age represents the seniority of a company, as measured by the number of years it has been in existence.

The operating conditions can promote or restrain the innovation of enterprises. In terms of operating conditions, we selected ROA (Return on Assets), Growth and Cash to control. ROA is the return on total assets of the company during the year, representing the company’s profitability. Growth refers to the growth rate of the company’s total assets during the year, and represents the company’s growth ability. Cash is the logarithm of the company’s monetary capital for the year, and represents the degree to which a company’s capital is constrained.

Corporate governance has an important impact on corporate behavior, which is reflected in R&D strategy. Therefore, in the model, to verify the impact of policies on innovation, corporate governance variables should be controlled. In terms of this, we selected Herfindahl10, Stock_hold and SOE (State-owned Enterprise) to control. Herfindahl10 represents equity concentration, which is embodied in the sum of squares of the top 10 major shareholders. Stock_hold refers to agency cost, measured by the number of shares held by senior executives in the firm divided by the total number of shares in the firm. When the senior management has high shareholding, the agency cost of the company is lower. SOE represents the nature of ultimate corporate control. The business objectives and strategies of state-owned enterprises and non-state-owned enterprises may be very different, while listed companies in China account for a high proportion of Chinese enterprises. In order to make the results more accurate, this paper also controls for this variable in the equation.

In addition, in order to control for the characteristics of the firm that do not change with time and the annual characteristics that do not change with the firm, we have added firm-fixed and year-fixed effects. Table 1 shows the specific variable definition.

### 3.4. Research Model

Based on the selected panel data, we construct the following model to verify the hypotheses. In the two equations, the explained variables are Innovation_investment and Innovation_patent, respectively. The explanatory variable DID represents whether the region where the company is located has become the pilot region for medical big data during that year. The control variables included in the equation respectively cover the basic information, operation status and governance of the company, as well as the fixed effect of the company and the fixed effect of the year.

Under the condition that other possible interference factors are controlled, the effect of the implementation of the big data medical policy on the company’s innovation is verified. Since the values of the explained variables are all restricted data greater than or equal to 0, we use the tobit model, which is more accurate than OLS (ordinary least squares) estimates. The following models are used to test Hypotheses 1 and 2, respectively, which are the basic models for the regression of this paper. The variables in the model correspond to the variable definition table.

*Innovation_investment_i,t_* = *α*_0_ + *β*_1_*DID_i,t_* + *β*_2_*Size_i,t_* + *β*_3_*Lev_i,t_* + *β*_4_*ROA_i,t_* + *β*_5_*Growth_i,t_* + *β*_6_*Age_i,t_* + *β*_7_*Cash_i,t_* + *β*_8_*Herfindahl10_i,t_* + *β*_9_*Stock_hold_i,t_* + *β*_10_*SOE_i,t_* + *Year fixed effect* + *Firm fixed effect* + *ε_i,t_*, (1)

*Innovation_patent_i,t_* = *α*_0_ + *β*_1_*DID_i,t_* + *β*_2_*Size_i,t_* + *β*_3_*Lev_i,t_* + *β*_4_*ROA_i,t_* + *β*_5_*Growth_i,t_* + *β*_6_*Age_i,t_* + *β*_7_*Cash_i,t_* + *β*_8_*Herfindahl10_i,t_* + *β*_9_*Stock_hold_i,t_* + *β*_10_*SOE_i,t_* + *Year fixed effect* + *Firm fixed effect* + *ε_i,t_*, (2)

In the above cross-section model, *i* stands for firm *i* and *t* stands for accounting period *t*. The other variables are referred to in Table 1. *α*_0_ is the intercept term of the equation, and *β* is the coefficient value of each variable, used to check the degree of correlation between independent variables and dependent variables. The year-fixed effect controls the interference of time series, and the firm-fixed effect controls the influence of individual specificity. *ε* is the random perturbation term in the panel equation. Empirical regression is carried out according to the above model. The empirical results will be introduced in the following article.

## 4. Results

### 4.1. Descriptive Statistical Analysis

Table 2 shows the results of the descriptive statistics. As can be seen from the second column, the number of samples of all other variables was 613, except for the small number of samples observed due to the missing value of Innovation_investment. The variances of Innovation_investment and Innovation_patent are both larger than the mean, indicating that the innovation level varies greatly between samples. In contrast, the range of Innovation_patent is larger, indicating that the difference in innovation output between different samples is significantly greater than the difference in their willingness to innovate. The root cause of this phenomenon calls for further study. In addition, we observed that, among the other control variables (except dummy variables), only Growth and Stock_hold had the coefficient of variation greater than 1, which also indicated that the dispersion degree of these two variables was relatively high. The Growth ability and the degree of executive equity incentive of different pharmaceutical companies were different.

### 4.2. Parallel Trend Hypothesis Testing

Prior to baseline regression, the approach of the Difference in Differences (DID) method requires a parallel trend test. Parallel trend is a basic hypothesis of DID. The core meaning is that, before the implementation of the policy, there was no significant difference in the dependent variable values between the experimental group and the control group, and the two groups had a parallel development trend. Only when the parallel trend hypothesis is satisfied can many unobservable interference factors be excluded to ensure the accuracy of quasi-natural experiments. The common parallel trend hypothesis test is the placebo test, which verifies that there is not a significant difference between the experimental group and the control group in the years before the policy is implemented. Table 3 shows the parallel trend hypothesis diagram of the two dependent variables in the paper. DID_2 is a dummy variable, assuming that the policy was implemented two years before the actual time (assuming the policy was implemented in 2014). If the sample belongs to the experimental group and the sample year is after 2014, DID_2 is 1, otherwise 0. DID_1 is a dummy variable, assuming that the policy was implemented one year before the actual time (assuming the policy was implemented in 2015). If the sample belongs to the experimental group and the sample year is after 2015, DID_1 is 1, otherwise 0. By replacing the variables DID with DID_1 and DID_2 and substituting them into equation1 and equation2, the table shows that the coefficients of DID_1 and DID_2 were insignificant, regardless of the presence or absence of control variables, and there was no significant difference between the control group and the control group before the experiment. Therefore, the placebo test was not significant, and did not prove a significant difference between the control group and the control group before the experiment. The parallel trend hypothesis is preliminarily verified.

### 4.3. Regression Analysis

Table 4 shows the results of the basic regression. In each set of validations, the uncontrolled variables (e.g., columns (1) and (3)) were first validated, followed by the controlled variables (e.g., columns (2) and (4)). It can be seen from the data in the table that the positive correlation between DIDs and Innovation_investment and Innovation_patent is both significant with or without the controlled variables. In other words, whether or not the control of the external environment is considered, the effect of policy implementation on the explained variables is very significant. In order to better control for the characteristics of each firm that do not change over time, we added the firm-fixed effect to the regression. At the same time, to control for year-to-year variability, we added the year-fixed effect to the regression. Compared with the control group, the Innovation_investment level and Innovation_patent level of the experimental group are significantly improved after the implementation of the big data policy. When considering the economic implications, we can see from the table that, in the case that the control environment was taken into account, the Innovation_investment value of the experimental group after policy occurrence significantly increased by 1.927 compared to the control group. Similarly, in the case that the control environment was taken into account, the Innovation_patent value of the experimental group after policy occurrence significantly increased by 3.270 compared to the control group.

### 4.4. Regression Grouped by Size

Many previous studies have revealed barriers to scale and strength in innovation, and the fact that large and small companies can behave very differently in R&D. Therefore, we further verified the impact of the big data medical pilot under different firm sizes. Big and small companies behave very differently under the same big data healthcare policy incentives. First, we define the size of the firm. When a firm is smaller than the average size, we define it as Minor, and when the firm is larger than the average size, we define it as Major. The heterogeneity test results for company size are shown in Table 5.

We propose the idea that innovation investment is directly influenced by both innovation risk and the level of technology. The impact of big data healthcare policies on small businesses is more likely to work through the mechanism of risk reduction. Studies have shown that less than 80 percent of R&D can be translated into actual product applications. Compared to large companies, due to their own less capital and limited financing channels, small companies have a lower risk level. The original high-risk aversion not only brings stability to small enterprises, but also weakens the utilization efficiency of social medical resources. In contrast, the construction of the big data platform can provide more complete drug categories and demands, more clear R&D (research and development) direction, and more accurate control of the application target group of R&D results. Lower risk levels can boost small firms’ willingness to engage in R&D, which is reflected in higher innovation spending on that topic. Although large companies also enjoy the benefits of risk aversion, they have a higher risk-bearing capacity, thus this effect is more significant in small companies.

In contrast, the impact of big data medical policy on the innovation patents of medical enterprises is mainly reflected in the level of technology. The big data platform can open the information barrier between medical institutions and realize more information exchange. However, at the same time, the dynamic, heterogeneous and huge nature of medical big data also puts forward higher requirements for technology when providing support for research. Whether in the emerging cross-industry technologies such as data mining and machine learning, or the existing basic medical technology in the industry, large enterprises are more mature and professional. After the construction of the big data platform, large enterprises can quickly rely on their own advantages, overcome data barriers and significantly improve the level of innovation. The heterogeneity test in Table 5 also preliminarily verified the above inference. In the performance of Innovation_investment, small companies (the group Minor) were more significant, while in the performance of Innovation_patent, large companies (the group Major) were more significant.

## 5. Discussion

Based on China’s big data medical pilot policy, this paper verified the positive effect of big data on medical innovation from an empirical perspective. It further proposes the heterogeneity of this effect, providing evidential support for the related theoretical research. In addition, big data medical treatment has high application value, and we suggest that it is necessary to build a comprehensive medical system based on big data. The following are a few related considerations.

The first discussion is about medical big data itself. Data, especially the broad range of big data, is crucial to the health care system, but there is some debate about the actual effect of big data on public health. This article provides empirical evidence of real-world application for further discussion in this area. It shows that the digitalization of information in the healthcare industry will generate a lot of application value, which will be linked to multiple stakeholders, not only at the economic level, but also with positive externalities on public health.

The second discussion is about big data medical and government intervention or guidance. This paper argues that government intervention or guidance is necessary. Stakeholders involved in the medical industry all have different interests and motivations. Although their behaviors affect and interweave with each other, the information pool that produces data is not integrated and recorded, and it is difficult for stakeholders to transform and collect the data on their own. This kind of governmental intervention is necessary for society to acquire data value.

The third discussion is on the heterogeneity of user size and the application of big data. Although every element of big data is relevant to public health, the emphasis of different elements seems to be quite different when applied. Individuals are providers of information and the beneficiaries of data products. Small companies or enterprises can rely on more comprehensive data for decision-making support, reduction of research costs, and lowered operational risks. Big companies and public institutions can make the most of data and leading the transformation of the industry.

The fourth discussion is on medical big data and big data property rights. There are no complete rules governing the generation, collection, analysis and application of data at this time. However, the clear property rights should be fully considered in the collection of medical big data. Ethics is the premise of all research; thus the property rights of data may also be an important direction of medical research regarding big data in the future.

Lastly, after verifying the policy effect of the experimental group, it is necessary to extend the policy effect to the control group. In the medical reform policy, the establishment of the diversified pattern of the big data medical system is increasingly promoted, and the significance of the pilot policy lies in the extension and promotion of the results. Although the pilot areas of big data have achieved initial results, the medical research and development process of local enterprises has been promoted. However, it still faces serious problems regarding unstructured data, lack of data coverage and the mismatch of data connection, which is especially prominent when data is merged in different places. Therefore, in the process of the policy promotion of the control group in the later stages, this paper suggests that regulators focus on making clear planning and unified definitions of the basic and necessary data of all regions in the early stages of medical big data construction, so as to realize cross-ground communication and telemedicine.

## 6. Conclusions

In terms of how to improve the level of public health comprehensively, the existing articles have reported on a very comprehensive study and mainly focus on the importance of funding and the transfer of benefits. From the perspective of the impact of science and technology, this paper introduces the exogenous event of China’s big data medical construction to explore the underlying subversive changes of science and technology to the R&D (research and development) of the medical industry. The results indicate that, after the implementation of the big data platform in the pilot provinces, the R&D willingness and level of local-listed companies have improved. Furthermore, the heterogeneity of this effect is related to firm size.

In addition, the article shows that the provision of data is conducive to finding more development information and direction, thereby reducing the risk of research and development, and thus the willingness to innovate is rapidly enhanced. In contrast, small companies have week financing and risk-aversion abilities, so their feedback on risk aversion is more obvious. While for large companies, incomplete data sets caused by industry competition limit the ceiling of R&D. After the platform makes up the shortage of information resources and breaks through the barrier of the data blockade, the big companies with superior original technology can get more “soft information” that they could not access before. Therefore, the innovation efficiency is improved based on the original technology.

The results of this paper provide an effective supplement to the theoretical framework of medical big data research, especially for the economic consequences and scientific and technological impact of big data medical research. Specifically, the main results support the construction of a government-led, big data medical platform, and suggest that it should be actively carried out in the control provinces in the future. Regarding the cost-benefit analysis, the economic externalities of platform construction and the specific issues of platform management are still being explored without a consensus being reached, which is a possible research direction in the future.

Moreover, from the perspective of logical deduction, the impact of medical big data on public health includes the micro level of individual, meso level of industry and macro level of society, and the influence path of these three levels may be different. In addition, the role of medical big data is not limited to cross-sectional effects, and it can be expanded into time series analysis in the future. Limited to the current data availability, this paper only takes the impact of big data on medical research and development as a starting point, and carries out the meso-level and cross-sectional effect test. Over time and with the accumulation of data, the authors plan to take a deeper research and carry out more detailed multi-layer analysis.

## Figures and Tables

**Table 1:** Variable definitions.

Variable	Variable Definition
**Dependent Variables**	
Innovation_investment	This variable represents the innovative actions of the enterprise. It is measured as the natural logarithm of R&D (Research and Development) expenditures disclosed in the financial statements.
Innovation_patent	This variable represents the innovative output of the enterprise. It is measured by the number of patents granted during the fiscal year.
**Independent Variables**	
DID	DID (Difference in Differences) is a dummy variable. If the province where the firm operates is the pilot place for medical big data and the data year is after the time that the policy is implemented, then the value of DID is 1; otherwise it is 0.
**Control Variables**	
Size	This variable is used to measure the size of a company. It is calculated by the natural log of the firm total assets, plus one.
Lev	Lev (Leverage) is used to measure a company’s debt level. It is calculated by the ratio of total liabilities divided by total assets.
Age	This variable mainly measures the age of the company, calculated by the years of firm establishment.
ROA	ROA (Return on Assets) mainly measures the profitability of the company. It is the net profit of the current year/total assets at the end of the year.
Growth	This variable mainly measures the growth status of the company. Growth = (Total assets this year, minus Total assets last year) × 100%/Total Assets last year
Cash	This variable mainly measures the capital constraint of enterprises, and it is the logarithm of the company’s monetary capital for the year.
Herfindahl10	This variable is used to measure ownership concentration in corporate governance. It is the sum of squares of the top 10 major shareholders.
Stock_hold	This variable is used to measure the agency cost of a company. When the proportion of senior executives’ shareholding is higher, the agency cost is lower. It is calculated by the number of shares held by senior executives in the firm/the total number of shares in the firm.
SOE	SOE (State-owned Enterprise) is the dummy variable that distinguishes property rights. It equals one if the firm is state owned, and zero otherwise.
Year-fixed effect	Year dummy variable, controlling the interference factors in time series.
Firm-fixed effect	Firm dummy variable, controlling the interference factors in cross section.

Notes: This variable definition table contains all of the variables in the baseline regression.

**Table 2:** Descriptive statistics.

Variable	Obs	Mean	Std. Dev.	Min	Max
Innovation_investment	395	8.437	8.500	0.000	21.437
Innovation_patent	613	5.162	13.932	0.000	152.000
DID	613	0.137	0.344	0.000	1.000
Size	613	21.756	1.079	18.532	25.567
Lev	613	0.332	0.194	0.026	0.941
ROA	613	0.071	0.048	0.000	0.267
Growth	613	0.277	0.495	−0.437	5.912
Age	613	16.522	4.909	6.000	36.000
Cash	613	19.900	1.127	15.827	23.652
Herfindahl10	613	59.337	14.066	19.360	100.000
Stock_hold	613	0.137	0.181	0.000	0.731
SOE	613	0.217	0.413	0.000	1.000

Notes: Obs is the sample observed number, Mean is the mean, Std. Dev. is the standard deviation, Min is the minimum, and Max is the maximum.

**Table 3:** Parallel trend hypothesis testing table.

Variable	Innovation_Investment		Innovation_Patent	
	**(1)**	**(2)**	**(3)**	**(4)**
DID_2	−0.754	−0.406	4.809	4.903
	(−0.607)	(−0.339)	(1.568)	(1.604)
DID_1	1.304	1.067	−0.507	−1.024
	(1.205)	(0.999)	(−0.208)	(−0.414)
Size		2.680 ***		2.877 *
		(3.124)		(1.921)
Lev		4.608 *		−4.980
		(1.763)		(−0.973)
ROA		12.899 *		6.180
		(1.769)		(0.441)
Growth		0.242		−0.033
		(0.650)		(−0.042)
Age		0.931 ***		−0.487
		(4.474)		(−1.318)
Cash		−0.194		−1.551 *
		(−0.407)		(−1.782)
Herfindahl10		−0.048		0.073
		(−1.588)		(1.218)
SOE		−1.600		−0.889
		(−0.719)		(−0.176)
Stock_hold		−2.520		10.078
		(−0.773)		(1.626)
Constant	12.976 ***	−55.266 ***	18.955 ***	−5.595
	(9.550)	(−4.197)	(5.602)	(−0.232)
Year	yes	yes	yes	yes
Firm	yes	yes	yes	yes
Observations	395	395	613	613
LR chi2	776.26	809.41	683.34	691.52

Notes: The superscripts ***, **, and * indicate significance at the 1%, 5%, and 10% levels, respectively.

**Table 4:** Regression analysis table.

Variable	Innovation_Investment		Innovation_Patent	
	**(1)**	**(2)**	**(3)**	**(4)**
DID	1.788 *	1.927 *	3.542 **	3.270 *
	(1.713)	(1.845)	(2.059)	(1.882)
Size		2.757 ***		2.636 *
		(3.221)		(1.765)
Lev		4.807 *		−4.896
		(1.855)		(−0.962)
ROA		12.816 *		5.147
		(1.765)		(0.367)
Growth		0.271		0.035
		(0.730)		(0.044)
Age		0.863 ***		−0.405
		(4.152)		(−1.129)
Cash		−0.118		−1.563 *
		(−0.249)		(−1.796)
Herfindahl10		−0.044		0.072
		(−1.453)		(1.207)
Stock_hold		−1.812		10.202 *
		(−0.556)		(1.651)
SOE		−1.323		−0.819
		(−0.595)		(−0.163)
Constant	13.096 ***	−57.439 ***	18.541 ***	−1.956
	(9.716)	(−4.378)	(5.520)	(−0.081)
Year	yes	yes	yes	yes
Firm	yes	yes	yes	yes
Observations	395	395	613	613
LR chi2	777.72	811.74	684.06	691.90

Notes: The superscripts ***, **, and * indicate significance at the 1%, 5%, and 10% levels, respectively.

**Table 5:** Regression grouped by size.

Variable	Innovation_Investment		Innovation_Patent	
	**Minor**	**Major**	**Minor**	**Major**
DID	3.360 **	1.515	−0.081	5.769 **
	(2.028)	(0.996)	(−0.024)	(2.163)
Size	4.855 ***	−0.247	3.709 *	7.818 **
	(3.658)	(−0.117)	(1.850)	(2.178)
Lev	1.563	8.460	−7.142	−1.902
	(0.500)	(1.522)	(−1.284)	(−0.168)
ROA	14.425 *	15.623	12.736	20.165
	(1.722)	(1.039)	(0.970)	(0.641)
Growth	0.388	−1.064 *	0.725	0.099
	(0.595)	(−1.691)	(0.636)	(0.072)
Age	0.625 *	1.426 ***	−0.654	−0.738
	(1.794)	(4.008)	(−1.501)	(−1.119)
Cash	0.422	−0.523	0.444	−3.607 *
	(0.781)	(−0.545)	(0.503)	(−1.943)
Herfindahl10	0.031	−0.051	−0.003	0.124
	(0.762)	(−0.989)	(−0.034)	(0.971)
Stock_hold	−0.724	1.741	−1.545	−32.258 *
	(−0.145)	(0.230)	(−0.230)	(−1.790)
SOE	0.707	−4.200	−2.308	2.620
	(0.223)	(−1.459)	(−0.431)	(0.325)
Constant	−109.362 ***	−0.266	−53.827	−75.420
	(−4.677)	(−0.009)	(−1.399)	(−1.298)
Year	yes	yes	yes	yes
Firm	yes	yes	yes	yes
Observations	210	185	319	294
LR chi2	493.00	407.18	307.05	407.41

The superscripts ***, **, and * indicate significance at the 1%, 5%, and 10% levels, respectively.
